# Determination of prevalence of subclinical mastitis, characterization of intra-mammary infection-causing bacteria, and antibiotic susceptibility in dairy camels in Jigjiga City, Somali region, Ethiopia

**DOI:** 10.3389/fvets.2024.1398118

**Published:** 2024-05-23

**Authors:** Mohamoud Mohamed Jama, Hassan Abdi Hussein, Ziad Abdulahi Darod, Abdullahi Adan Ahad

**Affiliations:** ^1^Livestock and Forage Directorate, Somali Region Pastoral and Agro-Pastoral Research Institute, Jigjiga, Ethiopia; ^2^College of Veterinary Medicine, Jigjiga University, Jigjiga, Ethiopia

**Keywords:** antibiotic sensitivity, antimicrobial resistance, camel, California mastitis test, *Staphylococcus aureus*, udder health, Jigjiga, Ethiopia

## Abstract

**Background:**

Subclinical mastitis in camels, an inflammation of the udder without visible signs, can reduce milk quality and raise bacteria levels. Regular monitoring of camel milk is crucial for consumer safety.

**Methods:**

A cross sectional study was conducted in Jigjiga city, Ethiopia to investigate the prevalence and characteristics of subclinical mastitis in she-camels. The study included 244 lactating she-camels from three privately-owned camel dairy farms, and a questionnaire survey was conducted with 60 camel owners.

**Results:**

The overall prevalence of subclinical mastitis in she-camels was 10.6% (26/244), with no significant difference among the studied dairy farms. Risk factors that influenced the result of California Mastitis Test (CMT) included age and udder and leg hygiene. The study revealed that *S. aureus* was the most prevalent bacterium among the isolated bacteria, with a prevalence rate of 34.5%. This was followed by *S. agalactiae*, *S. dysgalactiae*, and *Pasteurella multocida*, with prevalence rates of 29.8, 19.4, and 16.2%, respectively. Among the isolated bacteria, 84.5% were sensitive to Erythromycin, 60% to Streptomycin, 44.7% to Oxytetracycline, and 36.7% to Tetracycline. Interviews with camel owners revealed that 66.7% used mixed herd grazing methods and reported feed shortage. Treatment practices for sick camels included modern veterinary drugs, traditional medicines, or a combination of both. The owners of camel dairy farms did not maintain proper hygiene practices during milking, such as not using soap when washing hands.

**Conclusion:**

Addressing camel mastitis necessitates access to alternative drugs, comprehensive herder training, and enhanced management practices.

## 1 Introduction

The one-humped camels (*Camelus dromedarius*) demonstrate remarkable adaptation to arid and semi-arid environments, enabling them to not only survive but also thrive. They possess the ability to produce milk even in the midst of severe droughts, a crucial advantage over cattle, sheep, and goats, which often suffer high mortalities under such conditions. Consequently, dromedaries play a vital role in sustaining the livelihoods of pastoralists, particularly in the pastoral areas of Africa (1). Milk is a very nutritional food that is rich in carbohydrate, proteins, fats, vitamins, and minerals. However, milk and milk products of dairy cows and camel can harbor a variety of microorganisms and can be important sources of food borne pathogens to humans (2). Mastitis, a prevalent disease among camels, poses a significant threat to both the health of these animals and the economic well-being of the pastoralists. As it can result in substantial financial losses, combatting mastitis becomes paramount in safeguarding the livelihoods of pastoralists (3).

Mastitis can occur in two main forms: clinical and subclinical. Clinical mastitis shows visible signs and abnormalities in the animal’s udder and milk, while subclinical mastitis leads to reduced milk production without obvious symptoms (4). Diagnosing subclinical mastitis can be particularly challenging because it often lacks visible signs, making it easy for the condition to go unnoticed. This can lead to a prolonged presence of the disease within the herd, causing higher economic losses compared to clinical mastitis. Farmers may not be aware of the issue until it has already had a significant impact on milk quality and production. Therefore, early and targeted testing of the milk is essential to detect and address subclinical mastitis promptly, ultimately minimizing economic losses and ensuring the overall health and productivity of the herd (5).

In Ethiopia, the dairy industry faces significant obstacles due to various diseases associated with livestock farming practices, with mastitis being the primary concern. The effects of mastitis on this sector are extensive and encompass several areas. These include the temporary or permanent impairment of milk production capacity, a decline in milk quality, milk wastage resulting from antibiotic drug residues, increased expenses related to veterinary care and labor, a reduced productive lifespan of the animals, diminished value of meat after slaughter, and losses arising from reduced overall dairy product output (6).

Reports of sub-clinical mastitis in camels have been documented in various regions of Africa, including Egypt (7), Somalia (8), and Kenya (9). Very recent studies conducted in two pastoral districts in southern Ethiopia, reported a prevalence of sub-clinical mastitis in dairy cows, camels, and goats as 33.3, 26.3, and 25.0%, respectively; and quarter-level prevalence of sub-clinical mastitis in cows, camels and goats as 17.6, 14.5, and 20%, respectively (10). Sub-clinical mastitis in Ethiopia has been over-looked, with limited research and information available on its prevalence, geographic distribution, impact on milk quality, and related consumer risks. There is a need to study and understand the risk factors and negative effects of mastitis on milk and milk products to improve prevention and control measures (11). Particularly, there is very limited knowledge about sub-clinical mastitis in camels, including its causes and occurrence. However, cases of mastitis in camel have recently been reported in Ethiopia (12).

Limited information exists on the antimicrobial resistance (AMR) of pathogens in camel milk, but a study conducted in southern Ethiopia found that *S. aureus* isolates in camel milk were resistant to multiple drugs. The use of antimicrobials in dairy farms, particularly in food animal production, has been connected to an increased resistance to tetracycline in *S. aureus* and *E. coli* strains causing mastitis. This practice is widely acknowledged as a contributing factor to the development of antimicrobial resistance (10). The objective of this study was to determine the prevalence of camel sub-clinical mastitis and identify the associated risk factors. With great emphasis on isolation, characterization, and determination of antibiotic sensitivity of the bacteria causing sub-clinical mastitis in and around Jigjiga City, Fafan Zone, Somali Region, Ethiopia.

## 2 Materials and methods

### 2.1 Description of the study area

**Figure 1:**
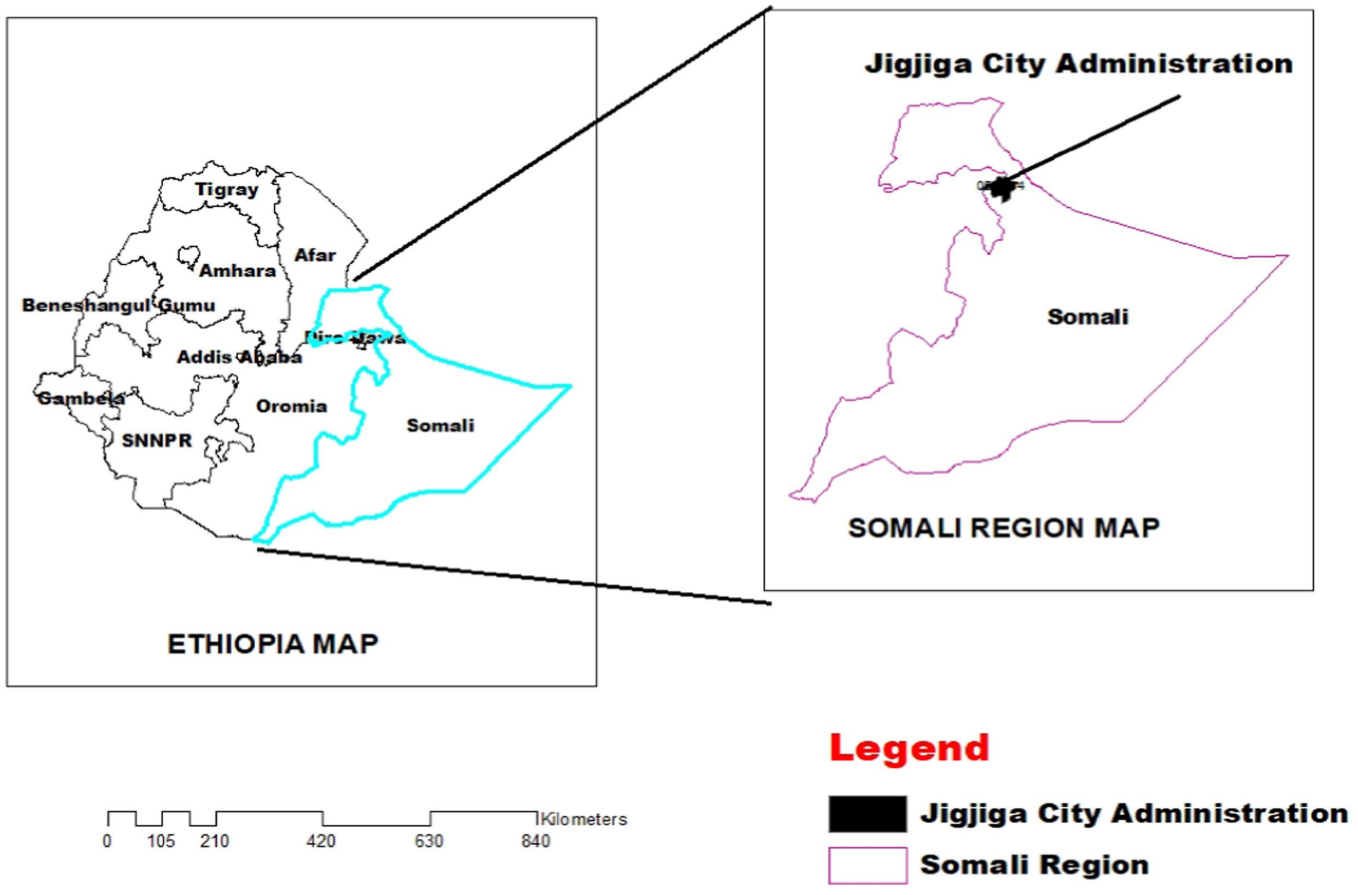
Map of the study area.

The study was conducted in and around Jigjiga city, Fafan zone, Somali regional state. Fafan administrative zone, is located in the northern part of the Ethiopian Somali Region, at 9°20′ North latitude; 45°56′° East longitude, about 630 km East of Addis Ababa. It covers a total land area of 40.86 km^2^ with altitude ranging from 500 to 1,650 m above sea level (masl). The area receives a precipitation ranging from 300 to 600 mm *per annum* and an average daily temperature of 16–20°C. Agro-pastoralist is a dominant production system in Fafan zone. The estimated livestock population of the zone is 248,435 cattle, 666,130 sheep, 503,881 goats 72,390 camels, and 10,548 poultry (13) (Figure 1).

### 2.2 Study design

A cross-sectional study was conducted from October 2021 to June 2022, on lactating female camels of local breed (*Camelus dromedarius*) reared either under intensive or extensive systems.

### 2.3 Study population

The study population consisted of lactating female camels from Jigjiga’s commercial dairy farms. The camels included in the study were all indigenous breeds of one humped camel (*Camelus dromedarius*) selected from three privately owned camel dairy farms (Dhaygel: *n* = 81, Barkomal: *n* = 131, Suleka: *n* = 32) that followed a semi-intensive farming system. Semi-intensive camel dairy farming in the Somali region combines aspects of intensive and extensive farming practices to optimize milk production from camels. This system includes regular milking, supplementary feeding, proper housing and management, breeding management, healthcare and disease management, as well as water and pasture management to ensure high-quality milk production while maintaining the health and wellbeing of the camels. The age of the animal was determined by examining dentation and owners records. In addition, A group of 60 specifically chosen camel owners, along with three owners/managers/attendants of camel dairy farms, were interviewed for the study.

### 2.4 Questionnaire survey

During the farm visits, data were collected using a pretested semi-structured questionnaire administered through personal interviews at each time. The information gathered included farm biodata and herd management practices of camel owners, such as the production system (pastoral or agro-pastoral), the purpose of keeping livestock (milk, meat, income, or social prestige), camel herd grazing methods (mixed with other species or grazing separately), cleaning the house (yes or no), milking practices (with or without calf suckling), washing hands before milking (yes or no), and cleaning milking equipment (yes or no).

### 2.5 Sample size determination

The sample size was determined using the formula described by Thrusfield (14), considering an expected prevalence of 18.1%, an absolute precision of 5%, and a 95% confidence interval. However, to increase precision, the sample size was increased to 244 lactating camels that were sampled in this study.


$$n=\frac{{\left(1.96\right)}^{2}P\left(1-P\right)}{d^{2}}\;n=\frac{{\left(1.96\right)}^{2}0.181\left(1-0.181\right)}{0.05^{2}}=228$$

Where: *P* = expected prevalence

*n* = required sample size

*d* = desired absolute precision

### 2.6 Sampling technique

First, the udder was cleaned to remove any dirt or debris. Following this, each teat was wiped with a clean cloth and disinfected using 70% alcohol. The initial streams of milk were discarded, and approximately 10–20 mL of milk was collected from each quarter (5–10 mL in each teat) in sterile universal bottles with unique labels. Risk factors, such as age, lactation stage, parity, production system, source of water, and udder and leg hygiene, were all documented.

### 2.7 California mastitis testing

To assess the clinical form of mastitis, milk from each quarter was examined using a strip cup, and any observable changes in color, odor, and consistency were recorded. Additionally, the presence of subclinical mastitis was determined using the California mastitis test (CMT) following the prescribed procedures outlined by Ferronatto et al. (15). The CMT results were interpreted subjectively according to the categories of negative, trace, 1+, 2+, or 3+, as outlined by Adkins and Middleton (16). Using the CMT, camels were classified as positive for SCM if they had readings of (1+, 2+, or 3+), while negative and trace readings were considered as negative.

### 2.8 Bacteriological laboratory examination and antimicrobial susceptibility testing

Bacteriological examination and AMR testing were conducted following the methods described by Quinn et al. (17) and the Clinical and Laboratory Standards Institute (CLSI) guidelines (18). For the analysis, a loopful of milk sample was streaked onto tryptose blood agar base supplemented with 5% sheep blood agar (Oxoid, United Kingdom) and MacConkey Agar using the quadrant streaking technique for each quarter. After incubation, the cultural growth macro-and micro-scale characteristics were evaluated, examining distinctions of each colony including colony morphology, hemolysis, and pigment production. Gram staining was performed on pure culture colonies to analyze their staining reaction and cellular morphology under a light microscope at 100× magnification. To avoid confusion in the Gram stain reaction, a potassium hydroxide (KOH) test was also conducted. For further examination, mixed colonies and Gram-negative bacteria were sub-cultured on sheep blood and MacConkey (Oxoid, Hampshire, United Kingdom) agar plates. Pure cultures of single colony types from both blood and MacConkey agar were transferred onto nutrient agar-medium for a series of primary tests including Catalase, Oxidase, Motility, and Fermentative-Oxidative tests, as well as secondary tests like triple sugar iron agar, citrate utilization test, methyl red test, and indole test, following standard procedures (19, 20).

Isolated bacterial colonies was transferred to a tube with sterile normal saline, creating a homogenous suspension adjusted to a turbidity equivalent to a 0.5 McFarland standard. The bacterial suspension were then inoculated onto Muller-Hinton agar using a sterile swab to cover the entire surface, with plates left to dry at room temperature. Prepared plates was checked for sterility and incubated overnight at 37°C before antimicrobial disks were placed on the media surface. A variety of commonly used antibiotics were selected for susceptibility testing in accordance with CLSI criteria. Standard antimicrobial impregnated disks (HiMedia Mumbai, India) were used, including Erythromycin 15 μg, Streptomycin 10 μg, Oxytetracycline 30 μg, and Tetracycline 30 μg. The zone of inhibition diameters around the disks were measured with rulers, and isolates were classified as susceptible, intermediate, or resistant following CLSI standards. Isolates resistant to three or more antimicrobial subclasses were considered multidrug resistant (21).

### 2.9 Data analysis

The data obtained from laboratory investigations and questionnaire survey were organized in a Microsoft Excel spreadsheet and analyzed using STATA (version 16; Stata Corp LP, College Station, TX, United States). Descriptive statistics were used to determine the prevalence of subclinical mastitis. The associations between subclinical mastitis and various factors (such as location/farm, age, lactation stage, parity, and production system, sources of water, and udder and leg hygiene) were evaluated using the chi-square test (χ^2^). The odds ratio (OR) was utilized to indicate the level of association between risk factors and SCM occurrence, with 95% confidence intervals provided. Variables with a *p* value <0.25 in univariate logistic regression analysis were included in a multivariate logistic regression analysis to control for potential confounding variables, and adjusted odds ratios were calculated. The model’s goodness-of-fit was assessed through backward elimination, where variables were sequentially removed starting from the least influential until the removal of a variable had a significant impact on the dependent variable. Collinearity between variables was checked using standard error, and model fitness was evaluated through the Hosmer and Lemeshow test and Omnibus test. A 95% confidence level was maintained throughout the data presentation, and a *p* value less than 0.05 (i.e., *p* < 0.05) was considered statistically significant.

## 3 Results

### 3.1 House hold questionnaires and dairy camel farm owners interview survey

**Figure 2:**
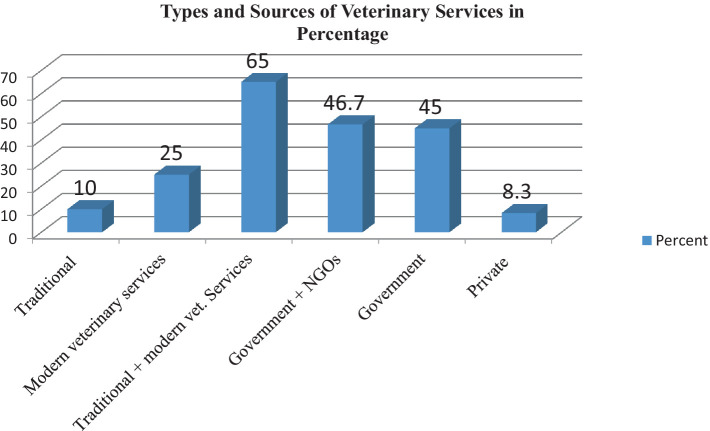
Category and sources of animal health service delivery system in the study area.

In the study area, the main feed sources for dairy camels were natural pasture and crop residue, as stated by 58.3% of respondents. Feed shortage problems were reported by 98.3% of dairy camel owners. Out of 60 respondents, 85% were aware of clinical mastitis in lactating she camels, but none were aware of subclinical mastitis in apparently health she camels. When treating mastitis, 65% used modern veterinary drugs and 25% relied on traditional medicines, particularly experienced camel owners. Most respondents (93.3%) had access to veterinary services. The summarized survey results are presented in Tables 1, 2, with a bar column chart (Figure 2).

**Table 1:** Dairy camel owners’ socio-economic profile and herd management.

Categories	Proportions (%)
Educational level	
Illiterate	45.0
Primary school	21.8
Religious knowledge	28.3
Secondary school	5.0
Livestock production system	
Pastoral	15.0
Agro—pastoral	85.0
Purpose of keeping camels	
Milk	60.0
Social and cultural values	10.0
Income generation	18.3
All the above purposes	11.6
Stage of lactation	
Early (First 3 months)	30.0
Mid (4–6 months)	45.0
Late (7 months onward)	25.0
Camel herd grazing methods	
Mixed with other animals	66.7
Grazing camels alone	33.3
Major feed sources for dairy camels	
Natural pasture	41.7
Natural pasture and crop residue	58.3
Feed shortage problems	
Yes	98.3
No	1.7
Source of water for camels	
Well	21.6
Dam/pond	51.6
River	18.3
Spring	8.3
Distance to watering point	
At home	5.0
Less than 1 km	13.3
More than 1 km	81.6
Frequency of watering	
Freely available	11.6
1 day interval	3.3
2 days interval	33.3
3 days interval	38.3
Once in a week	13.3
Housing system	
Together with other spps	66.7
Separately alone	33.3
Do you clean camel house	
Yes	81.7
No	18.3

**Table 2:** Major constraints of camel milk production and handling practices in three dairy farm camels in Somali region, Ethiopia.

**Categories**	**Proportion (%)**
Major constraints to she camel milk production	
Lack of Vet. Services	13.33
Fodder and Vet. Service	18.33
Shortage rangeland and vet. Services	65
Marketing, shortage of rangeland and vet. Service	3.34
Type of milking practices	
Few suck before milking	81.7
Milk without suck	18.3
Where do you milk the camel	
Outside the barn	78.3
Inside the barn	21.7
Washing hands before and after milking	
Yes	75
No	25
Washing with Soap	
Yes	16
No	84
Types of materials used for milking	
Wood container	68.3
Plastic container	15
Both	16.7
Purpose of camel milk production	
Selling	41.7
Home consumptions	3.3
Both of the above	55
Do you clean the milking equipment regularly	
Yes	96.7
No	3.3
How to consume camel milk	
Raw	96.7
Fermented state	3.3

### 3.2 Prevalence of subclinical mastitis

In the study area, the overall prevalence of subclinical mastitis in she-camels was 10.6% (95% CI: 0.067–0.146) with 26 out of 244 individuals affected. Among the three camel dairy farms examined, Suleka had the highest prevalence at 12.5% (95% CI: 0.003–0.246), followed by Dhaygel and Barkomal with prevalence of 11.1% (95% CI: 0.041–0.181) and 9.9% (95% CI: 0.047–0.151), respectively. However, there were no significant differences in the prevalence of subclinical mastitis among the different dairy farms (*p* > 0.05) (Table 3).

**Table 3:** Prevalence of sub-clinical mastitis in the she-camels from the three dairy farm camels in Somali region, Ethiopia.

Camel dairy farms	Number of examined	Number of positives	Prevalence (%)	χ^2^	*p*-value
Dhaygeel	81	9	11.1		
Barkomal	131	13	9.9		
Suleka	32	4	12.5	0.20	0.90
Total	244	26	10.6		

She camels in the 5–7 years age category were found to be at a greater risk compared to those aged 2–4 and over 7 years. Poor hygiene of the udder and legs were identified as significant risk factors for subclinical mastitis in she-camels, with poor udder and leg hygiene being the most affected factors (*p* < 0.05). However, factors such as location/farm, lactation stage, parity production system, and sources of water for the farms did not display a statistically significant association with the occurrence of subclinical mastitis in the study areas (*p* > 0.05) (Table 4).

**Table 4:** Multivariable logistic regression analysis of factors associated with subclinical mastitis in lactating camels from three dairy farms in the Somali region of Ethiopia.

Risk factors	No of examined	No of positive, *n* (%)	OR [95%CI]	χ^2^	*p*-value
Location/Farm					
Barkomal	131	13 (9.9)			
Dhaygel	81	9 (11.1)	1.1		
Suleka	32	4 (12.5)	1.3	0.2	0.904
Age					
2–4 years	132	7 (5.3)			
5–7 years	36	13 (36.1)	10.1		
>7 years	76	6 (7.8)	1.5	21.8	0.0000***
Lactation stage					
Early	144	17 (11.8)			
Late	100	9 (9)	0.73	0.5	0.48
Parity					
Few (1–3)	162	14 (8.6)			
Many (≥4)	82	12 (14.6)	1.8	1.97	0.16
Production system					
Extensive	113	13 (11.5)			
Semi-intensive	131	13 (9.9)	0.84	0.16	0.69
Source of water					
Tap water	32	4 (12.5)			
Well	212	22 (10.4)	0.81	0.13	0.722
Udder and leg hygiene					
Good	170	1 (0.5)			
Medium	51	7 (13.7)	26.8	88.41	
Poor	23	18 (78.2)	608.4		0.0000***

### 3.3 Bacterial species isolation analysis

In the 26 cultured samples, a total of 510 bacterial colonies were found. The most common species was *Staphylococcus aureus*, comprising 34.5% of the isolated bacteria. Other bacteria like *Streptococcus agalactiae*, *Streptococcus dysgalactiae*, and *Pasteurella multocida* were also present, with proportions of 29.8, 19.4, and 16.2%, respectively (Table 5).

**Table 5:** Distribution of isolates and individual prevalence of bacterial species isolated from she camels in three dairy farm camels in Somali region, Ethiopia.

Name of the farm	*Staphylococcus aureus, n (%)*	*Streptococcus agalactiae, n (%)*	*Streptococcus dysgalactiae, n (%)*	*Pasteurella spp., n (%)*
Barkomal	31 (6.1)	46 (9.0)	27 (5.2)	0 (0)
Dhaygel	80 (15.6)	51 (10.0)	29 (5.6)	40 (7.8)
Suleka	65 (12.7)	55 (10.7)	43 (8.4)	43 (8.4)
Total	176 (34.5)	152 (29.8)	99 (19.4)	83 (16.2)

### 3.4 Antibiotic sensitivity test (Kirby-Bauer disk diffusion method)

The study found that *Staphylococcus*, *Streptococcus*, and *Pasteurella* species isolates had higher susceptibility to erythromycin compared to other antibiotics tested. Erythromycin was the most effective antibiotic, with high susceptibility among the identified bacterial species. Streptomycin was the second most effective antibiotic. In contrast, oxytetracycline and tetracycline antibiotics showed low sensitivity among the identified bacterial species. The resistance profile against tetracycline was relatively high among the bacteria tested (Table 6).

**Table 6:** *In vitro* susceptibility test of bacterial profiles from camel milk samples with subclinical mastitis in somali region dairy farms.

	*Staphylococcus aureus*		*Streptococcus agalactiae*		*Streptococcus dysgalactiae*		*Pasteurella multocida*		Total	
Antibiotics	*N*	%	*N*	%	*N*	%	*N*	%	*N*	%
Erythromycin	176/176	100	136/152	89.4	61/99	61.6	58/83	69.8	431/510	84.5
Streptomycin	93/176	52.8	110/152	72.3	54/9	54.5	49/83	59	306/510	60
Oxytetracycline	65/176	36.9	76/152	50	45/99	45.5	42/83	50.6	228/510	44.7
Tetracycline	54/176	30.6	66/152	43.4	34/99	34.3	33/83	39.8	187/510	36.7

*N*, Number of sensitive bacteria/evaluated number of bacteria.

## 4 Discussion

The prevalence of subclinical mastitis in she camels in this study (10.6%) aligns with previous studies by Mohamud et al. (8) who reported 9.8% in Somalia, and Juboori et al. (22) who reported 11.67% in the UAE. Similarly, the findings are comparable to Balemi et al. (10) who reported 14.5% in Ethiopia. However, Geresu et al. (12) found a slightly higher prevalence of 18.1% in Southern Ethiopia. On the other hand, the camel mastitis prevalence found in the present study is relatively lower than the reported 59.8% in the Afar Region of Ethiopia (23), 76% in pastoral areas of eastern Ethiopia (24), 18.5% in Abu Dhabi, United Arab Emirates (25) and 34.7% in Borena zone of Oromia Regional State (26). Contrastingly, Almaw and Molla (27) reported a lower incidence of 2.1% for subclinical mastitis in lactating she camels in northeastern Ethiopia, which is below the prevalence indicated in the current study. The higher prevalence of those research studies may be attributed to differences in the management systems of camels.

The 5–7 years age group showed a significant difference in subclinical mastitis compared to other age groups, aligning with previous studies on the correlation between age of she camels and sub-clinical mastitis prevalence by Geresu et al. (12), which found a significant difference in sub-clinical mastitis among different age groups (*p* < 0.05). Additionally, our study found that udder and leg hygiene measures significantly influenced the prevalence of camel subclinical mastitis. Nevertheless, lactation stage is not significantly associated with the occurrence of camel mastitis in the study areas, with (*p* > 0.05) consistent with the findings of Mahboob et al. (28).

In addition, Alebie et al. (29) found that neither parity nor lactation stages were statistically significant in the occurrence of camel subclinical mastitis in lactating she camels from Dubti district, Afar Regional State, Northeastern Ethiopia, which is consistent with our current results. However, Mogeh et al. (30) reported a significant difference in camel subclinical mastitis across lactation stage and parity (*p* < 0.05) among lactating dromedary camels in and around Hargeisa, Somaliland. Variation in research methodologies and types of bacteria examined across previous studies may contribute to the differences in findings and discrepancies in reported rates of subclinical mastitis.

The findings of the study regarding the respondents’ knowledge and practices related to mastitis in she camels are worth discussing. It is noteworthy that a significant majority of respondents (85%) were aware of clinical mastitis in lactating she camels, indicating a basic understanding of this condition. However, the complete lack of familiarity with subclinical mastitis in seemingly healthy she camels among the respondents highlights a gap in knowledge that could potentially impact the health management of the camel herd. The treatment practices reported by the respondents also provide insights into the current approaches taken in managing mastitis. The fact that 65% of respondents turned to modern veterinary drugs for treating mastitis suggests a reliance on evidence-based interventions. On the other hand, the 25% of respondents who preferred traditional medicines and sought advice from experienced camel owners indicate a potential reliance on indigenous knowledge and practices. This brings to light the blending of modern and traditional approaches in managing mastitis in she camels within the community. Furthermore, the high percentage of respondents (93.3%) having access to veterinary services is an encouraging finding as it indicates the potential for professional guidance and support in managing mastitis cases. This accessibility to veterinary services can contribute to improved diagnostic and treatment outcomes for mastitis in she camels. These findings are consistent with the report by Seligsohn et al. (9).

The study findings indicate that a majority of farmers (75%) claim to wash their hands before milking. However, an even larger proportion (96.7%) regularly clean the milking equipment. It is worth noting that a significant number of farmers (84%) do not use soap during handwashing. This highlights a potential gap in hygiene practices that may require further attention and education among farmers. The absence of proper hygiene standards during milking processes could be a contributing factor to the spread of subclinical mastitis in camel herds Wang et al. (31).

The prevalence of *Staphylococcus aureus* and *Streptococcus agalactiae* in the current study, at 34.5 and 29.8% respectively, aligns with the results of Wubishet et al. (26), who reported 38.0 and 27.5% prevalence rates of *Staphylococcus aureus* and *Streptococcus agalactiae* isolates in mastitis-positive lactating she camels from the Borena Zone, Oromia Regional State, Ethiopia. Nonetheless, Compared to the 34.5% found in the current study, Eyassu and Bekele (32) reported a significantly lower percentage of *Staphylococcus aureus* (4.2%).

The present study has shown a high level of multi-drug resistance against commonly used drugs such as Tetracycline and Oxytetracycline. However, Erythromycin was found to be the most effective antibiotic, with high susceptibility among the isolated bacterial species, this is in line (33). Based on the results of the antibiotics sensitivity test in the current study, it can be concluded that the preferred antimicrobial drugs for treating mastitis in dairy camels should be Erythromycin first, followed by Streptomycin and Oxytetracycline in descending order.

## 5 Conclusion

The present study revealed a high prevalence of sub-clinical mastitis in dairy camels, indicating it as a significant health problem. Among the risk factors considered, age and hygienic measures such as udder and leg hygiene showed a significant association with the prevalence of sub-clinical mastitis in lactating she camels. The major bacterial species identified as the cause of mastitis in the study area were *Staphylococcus aureus*, *Streptococcus agalactiae*, *Streptococcus dysgalactiae*, and *Pasteurella multocida*. The antibiotic sensitivity test indicated that Erythromycin was the preferred drug for treatment, while Oxytetracycline and Tetracycline showed the least efficacy in the study area. Based on the findings, it is recommended to focus on various aspects to address camel mastitis. These include conducting further research, utilizing traditional knowledge, increasing the availability of alternative drugs, providing comprehensive training, and implementing improved management practices. These measures aim to reduce the prevalence and transmission of the disease.

## Data availability statement

The raw data supporting the conclusions of this article will be made available by the authors, without undue reservation.

## Ethics statement

The animal studies were approved by ethics committee of Somali region pastoral and agro-pastoral research institute (SORPARI). The studies were conducted in accordance with the local legislation and institutional requirements. Written informed consent was obtained from the owners for the participation of their animals in this study.

## Author contributions

MJ: Conceptualization, Data curation, Formal analysis, Funding acquisition, Investigation, Methodology, Project administration, Resources, Supervision, Validation, Visualization, Writing – original draft, Writing – review & editing. HH: Data curation, Formal analysis, Funding acquisition, Investigation, Methodology, Resources, Validation, Visualization, Writing – original draft, Writing – review & editing. ZD: Formal analysis, Investigation, Methodology, Writing – original draft, Writing – review & editing. AA: Data curation, Investigation, Methodology, Writing – original draft, Writing – review & editing.
